# From predisposition to recovery: field evidence of interactions between the gut microbiota and *Brachyspira hyodysenteriae* infection

**DOI:** 10.1186/s13567-025-01646-1

**Published:** 2026-01-30

**Authors:** Lucía Pérez-Pérez, Héctor Arguello, José F. Cobo-Díaz, Cristina Galisteo, Héctor Puente, Samuel Gómez-Martínez, Ana Carvajal

**Affiliations:** 1 Departamento de Sanidad Animal, Facultad de Veterinaria, Universidad de León, León, Spain; 2 INDEGSAL, Universidad de León, León, Spain; 3 Departamento de Higiene y Tecnología de los alimentos, Facultad de Veterinaria, Universidad de León, León, Spain; 4 Departamento de Anatomía y Anatomía Patológica Comparadas y Toxicología, Universidad de Córdoba, Córdoba, Spain

**Keywords:** Swine dysentery, pig, shotgun metagenomics, intestine, spirochaetes

## Abstract

Restrictions on antibiotics use have increased interest in the gut microbiota relationship to host health, particularly in enteric infections. The present field study, performed on two farms with endemic swine dysentery (SD) infection, characterises the faecal microbiota in 102 faecal samples from 13 diseased and 13 non-diseased pigs by shotgun metagenomic sequencing. The samples were collected during four samplings, which allowed us to monitor the animals before, during and after the clinical disease to investigate the role of the gut microbiota in disease outcome, assess the impact of infection on microbial composition and evaluate the microbiota evolution following recovery. Samples collected before disease demonstrated that SD susceptible pigs had lower microbial diversity, with significantly lower abundance of *Treponema rectale*, *Prevotella* spp. or *Ruminiclostridium* E compared with SD resistant pigs, which remained healthy. Marked alterations in microbial species composition and their functional profiles were evident during clinical disease.* Brachyspira hyodysenteriae*, *Dysosmobacter* sp. BX15, *Acetivibrio* *ethanolgignens* and *Mucispirillum* sp. 910586745 were significantly increased in abundance, which was associated with an increase of functions such as *Bacteroides* capsular polysaccharide transcription antitermination proteins or pterin carbinolamine dehydratase. No changes in the microbiota were observed after the disease when compared with non-diseased pigs, thus evidencing a restoration of the microbiota composition after therapeutic treatment and recovery. The study demonstrates that the microbiota may play a relevant role in SD disease outcome and evidences the changes that occur during clinical disease do not persist over time after pig therapeutic treatment.

**Supplementary Information:**

The online version contains supplementary material available at 10.1186/s13567-025-01646-1.

## Introduction

The increased incidence of enteric infectious diseases in recent years linked to restrictive policies in the use of antibiotics [1, 2] has turned the spotlight to concepts such as gut health in animal production. The gut health involves an efficient nutrient digestion and absorption, a balanced status of the gut immune system and among other factors, the assembly of the commensal gut microbiota. Indeed, in recent years, the importance of the microbiota in gut health has gained attention in different animal species such as *Sus scrofa* [3]. The microbiota is involved in multiple inter-bacterial and host–microbe interactions, playing a crucial role in pig health and productive performance [4], for instance, impacting feed conversion efficiency [5], the quality and composition of meat products [6] and, consequently, production profitability. Several studies have described how the microbiota influences key functions, such as pathogen protection through immune responses and intestinal barrier [7, 8]. The porcine gut microbiota exhibits a complex organization, with a dynamic composition and diversity along the gastrointestinal tract and production stages [9, 10]. This composition is also affected by external factors such as diet [11], environment [12], animal genetics [13] or infections [14].

Swine dysentery (SD), primarily caused by *Brachyspira hyodysenteriae*, is a severe enteric disease, characterized by mucohaemorrhagic diarrhoea and noticeable inflammation of the large intestine [15, 16]. It is a re-emergent disease in industrial pig production with new aetiological species such as *Brachyspira suanatina* and *Brachyspira hampsonii* [17, 18] and is further complicated by the reduced susceptibility to antimicrobials used in therapeutics [19]. A complex interaction between the microbiota in the large intestine and the aetiological agent was proposed decades ago [20]. Recently, several studies have expanded the knowledge of microbiota alterations linked to SD by high-throughput sequencing metagenomics [21–23]. These studies were performed using experimental infections and focused on microbiome alterations early after the onset of clinical disease. However, there is room for research from other perspectives, for instance, by gathering information from field studies with natural SD outbreaks, particularities and bottlenecks of SD challenge-models are bypassed. Such studies may provide valuable insights, for instance, into pathogen–microbiota interactions that influence disease progression and remain unclear [24].

Considering these premises, the present study analyses the impact of SD on the composition and functionality of the gut microbiota at three time points: before disease, during clinical disease and after recovery, on two farms with endemic *B. hyodysenteriae* infection, by using shotgun metagenomics. The main goals of the study focus on the analysis of the role of the microbiota in the SD susceptibility, the impact of natural infection in the microbiota composition and the evolution of the microbiota after pig recovery, providing novel insights into the interaction between *B. hyodysenteriae* and pig microbiota.

## Materials and methods

### Farms characteristics, swine dysentery monitoring and sample processing

The study was conducted on two Spanish extensive pig farms (hereafter named farm A and farm B) with an endemic history of SD. The disease was confirmed on both farms by the detection and isolation of *B. hyodysenteriae* in clinical faecal samples submitted to our laboratory following the protocol already described elsewhere [16, 25]. The antimicrobial resistance profile for each isolate was determined using commercial plates (VetMIC-Brachy, SVA, Sweden) and the procedure described by Vega et al. 2022 [26]. Feed was supplied to both farms by the same local feed company and feed composition used during the study period can be revised in the Additional file 1.

Two batches of pigs were monitored over a 6-month period, one batch of 99 pigs on farm A and another batch of 83 pigs on farm B. Each batch was sampled at four time points and was visited weekly by the farm veterinarian. Animals were individually ear-tagged at the first sampling (6–8 weeks of age), while samplings 2–4 were performed after the onset of the SD outbreak. The onset of the outbreak was defined by the observation of an animal with clinical SD by the farm veterinarian and the farmer (Table 1). On each sampling, individual faecal samples were aseptically collected and snap-frozen in dry-ice immediately after collection. Additional fresh faecal samples were collected from animals suffering diarrhoea and sent to the lab under cooling conditions for *B. hyodysenteriae* detection. Diseased animals were parenterally treated, within the first 36 h after the onset of SD signs, with lincomycin (LIVISTO, Germany) according to the product guidelines and the recommendations of the veterinarian visiting the farm. Samples were sent straight after their collection to the Infectious Diseases Unit of the Department of Animal Health at the University of León (Spain), where fresh samples were immediately processed while frozen samples were stored at –80 ℃ until DNA extraction.

**Table 1:** **Diagnosis of *B. hyodysenteriae*** **in faecal samples**

Pig	Farm	Date	Sampling	Culture	PCR
122	Farm A	06/05/2021	Additional^a^	Positive	Positive
139	Farm A	29/07/2021	Sampling 3	Positive	Positive
143	Farm A	29/07/2021	Sampling 3	Positive	Positive
385	Farm A	29/07/2021	Sampling 3	Positive	Positive
396	Farm A	29/07/2021	Sampling 3	Positive	Positive
422	Farm A	30/04/2021	Additional^a^	Positive	Positive
424	Farm A	06/05/2021	Additional^a^	Positive	Positive
430	Farm A	20/05/2021	Sampling 2	Positive	Positive
251	Farm B	15/06/2021	Additional^a^	Positive	Positive
300	Farm B	18/05/2021	Sampling 1	Positive	Positive
304	Farm B	26/05/2021	Additional^a^	Positive	Positive
305	Farm B	04/08/2021	Sampling 3	Positive	Positive
309	Farm B	26/05/2021	Additional^a^	Positive	Positive

^a^Additional sampling refers to samples collected in animals with clinical SD between samplings in the monitoring period.

### *Brachyspira hyodysenteriae* isolation and identification

Briefly, fresh faecal samples were cultured on trypticase soy agar (TSA, Scharlab, Spain) supplemented with 5% sheep blood (Oxoid, Spain), 400 μg/mL spectinomycin, 8 μg/mL colistin and 20 μg/mL vancomycin (Sigma-Aldrich, USA) and incubated under anaerobic conditions at 40 ℃ for up to 7 days. DNA was obtained from the *β*-haemolytic growth by freezing and used as template for PCR detection of the *B. hyodysenteriae* haemolysin regulatory gene *tlyA* [27].

### Sample selection and DNA extraction

From the frozen faecal sample collection obtained, samples from animals with confirmed SD and samples from non-diseased animals from the same batch were grouped in two subsets. Non-diseased animals were defined as pigs that remained negative to *B.* *hyodysenteriae* during the period of study.

DNA extraction from frozen faecal samples was performed using the QIAamp^®^ PowerFecal^®^ Pro DNA Kit (Qiagen, Germany) following the manufacturer instructions. The extracted DNA was quantified using the Qubit BR Assay (Thermo Fisher Scientific, USA) and stored at –80 ℃ until further analysis.

### Library preparation sequencing and bioinformatics analysis

Paired-end sequencing libraries were prepared from the extracted DNA using the Illumina Nextera XT Library Preparation Kit (Illumina Inc., USA) and sequenced on the Illumina NovaSeq 6000 platform (Novogene, UK) with 150 bp paired-end approach and a depth of 3 Gb per sample, following the manufacturer instructions.

Adapter removal and quality trimming of raw reads were performed using TrimGalore v.0.6.0 with default parameters [28], a wrapper script for Cutadapt v.2.6 [29] and FastQC v.0.11.8 [30]. Reads that mapped to the human (GRCh38) and pig (Scrofa 11.1) reference genomes were removed using Bowtie2 v.2.3.4.3 [31] with default parameters. The resulting BAM files were processed using SAMtools v.1.9 [32] and converted to FastQ format using BEDTools v.2.27.1 [33]. Taxonomic assignment of filtered reads was performed using MetaPhlAn 4 [34] with vOct22_CHOCOPhlAnSGB_202212 database and functional annotation was conducted with SUPER-FOCUS v.0.35 [35] using the 90 cluster size database. The full datasets have been submitted to the NCBI Sequence Read Archive (SRA) and are available under BioProject accession number PRJNA1218713.

### Statistical analysis

Comparisons among the monitored animals and through the samplings performed were analysed as follows. First, pigs were categorised as diseased and non-diseased on the basis of the detection and identification of *B. hyodysenteriae* in faeces (variable defined as disease). In addition, another variable (dysentery) was created to establish if, samples from diseased animals were collected before disease (pre-SD), at the SD clinical disease (clinical-SD), or after recovery (post-SD).

Samples metadata information, including farm (farm A and farm B), sampling time point (sampling 1 to sampling 4) and the two variables about the SD disease defined above are summarised in Additional file 1.

Based on the two SD variables, we explored changes in microbiota composition and functionality by comparing the metagenomics data from diseased pigs with counter-part samples from non-diseased pigs within the same sampling time point. The identification of taxa, which could play a role for predisposing/preventing SD, was explored by comparing the microbiota composition from sampling 1 and from the sampling previous to clinical disease (pre-SD samples). Potential changes in the microbiota associated with the onset of mucohaemorrhagic diarrhoea were assessed by comparing clinical-SD samples with counter-part samples collected at the same time point. Finally, the post-clinical assembly of the microbiota was evaluated by establishing taxonomic and functional differences in post-SD samples and in the final sampling (sampling 4), as well as comparing pre-SD and post-SD samples in diseased pigs and the respective samples at the same time point in the non-diseased group.

Metataxonomic and functional data analyses were performed in R v.4.3.3 [36]. Alpha and beta diversities were calculated from species and functional relative abundance data using the *vegan* v.2.6–4 R package [37] and visualized with *ggplot2* v.3.5.1 R package [38]. Alpha diversity was assessed using the Richness, Shannon, Pielou and Simpson indexes. The normality of each index was evaluated using the Shapiro Wilk test (*stats* v.4.3.3 R package [36]) and compared using one-way analysis of variance (ANOVA), followed by pairwise comparisons with the Tukey test (*stats* v.4.3.3 R package [36]) when data were normally distributed. If non-parametric, the Kruskal–Wallis test was applied, followed by pairwise comparisons using the Wilcoxon test (*ggpubr* v.0.6.0 R package [39]). *P*-values were adjusted using the Holm method.

Sample ordination was performed using Non-metric Multidimensional Scaling (NMDS) based on pairwise Bray–Curtis distances. The association between the variables studied and bacterial composition and functionality was tested using permutational multivariate analysis of variance (PERMANOVA) with the *adonis2* (*vegan* v.2.6–4 R package [37]) and *pairwise.adonis* (*pairwiseAdonis* v.0.4.1 R package [40]) functions. Intra-group dispersion was assessed by estimating the distances of each sample to its group centroid. Differences in dispersion were tested following the same statistical approach as for alpha diversity analysis. The influence of the 30 species and functions with the highest mean abundance on ordination was evaluated by fitting linear models to ordination results using the *envfit* function (*vegan* v.2.6–4 R package [37]), with Benjamini–Hochberg (BH) correction for multiple testing.

Differential species and function abundance was analysed using the linear models for differential abundance (LinDA) approach [41], implemented in the *MicrobiomeStat* v.1.2 R package [42] as the *linda* function. Species and functionality data with fewer than three non-zero values were excluded. Farm and sampling time point variables were included as covariates in the corresponding analyses, and significant species and functions were identified using a BH-adjusted *P*-value ≤ 0.05.

Community profiles were estimated for species constituting the core microbiota, defined as those detected at a threshold of 0.1% in at least 70% of the samples, using Dirichlet multinomial mixtures (DMM) modelling [43] (*DirichletMultinomial* v.1.44.0 R package [44]). Laplace approximation was used to evaluate DMM models fit, and the optimal number of components (clusters) was determined based on the lowest Laplace value. The main species driving differences between community types were identified as those with cluster contribution values above the 80^th^ percentile. Results were visualized by colouring samples in ordination plots according to cluster group, and differences between clusters were assessed using PERMANOVA [37, 40].

Partial Least Squares Discriminant Analysis (PLS-DA) was used to maximize the separation of predefined groups, identifying the most relevant species and functions contributing to their differentiation (*mixOmics* v.6.26.0 R package [45]). To identify potential associations, Spearman correlations were calculated between *B. hyodysenteriae* and the functional features, as well as between species and functions identified by PLS-DA and LinDA [46]. Results were filtered using a threshold of rho > 0.5 or < −0.5 and *P* ≤ 0.05.

## Results

### Animal and sample selection for microbiota analysis according to SD diagnostic outcomes

Swine dysentery was confirmed in fresh faecal samples from 13 pigs (8 from farm A and 5 from farm B) by microbiological culture and PCR identification of *B. hyodysenteriae* (Table 1). Results of antimicrobial characterization of the recovered isolates are shown in Table 2. Therefore, a total of 102 samples from 26 animals, 13 diseased and 13 non-diseased, and four sampling rounds were selected for metagenomic shotgun sequencing. In total, 16 of animals were from farm A and 10 from farm B.

**Table 2:** **Antimicrobial susceptibility of *B. hyodysenteriae*** **isolates from both farms**

Farm	Antibiotic	MIC (μg/ml)	Interpretation^a^
Farm_A	Tiamulin	< 0.06250	Susceptible
	Valnemulin	< 0.03125	Susceptible
	Doxycyclin	0.25000	Susceptible
	Tylvalosin	2	Intermediate
	Lincomycin	32	Intermediate
	Tylosin	> 128	Resistant
Farm_B	Tiamulin	2	Intermediate
	Valnemulin	1	Intermediate
	Doxycyclin	1	Intermediate
	Tylvalosin	8	Intermediate
	Lincomycin	16	Intermediate
	Tylosin	> 128	Resistant

MIC: Minimum inhibitory concentration

^a^Interpretation according to Vega et al. [26].

### Microbiota predisposing to swine dysentery

A comparative analysis of the microbiota from diseased and non-diseased pigs in sampling 1 revealed no significant taxonomic or functional differences between groups (Additional files 2, 3, 4, 5, 6, 7 and 8). However, a trend was observed (*P* = 0.087) towards higher species diversity in non-diseased pigs, as indicated by the Shannon index results (Additional files 2 A and 4). We explored the potential differences in this sampling 1 by farm as beta diversity analyses highlighted the farm of origin as the main driver of sample ordination (*P* < 0.001) (Additional files 6^6^and 7). Farm comparison evidenced higher species richness on farm B compared with farm A (*P* < 0.01) (Additional files 2A and 4). Interestingly, the analysis of samples from farm B, revealed that samples from pigs that subsequently became diseased displayed greater dispersion (*P* < 0.001) and lower abundance of *Roseburia* sp. 499 (log_2_FC = −6.01; *P* < 0.01) (Additional files 2, 3 and 8) compared with samples from their non-diseased counterparts.

**Figure 1:**
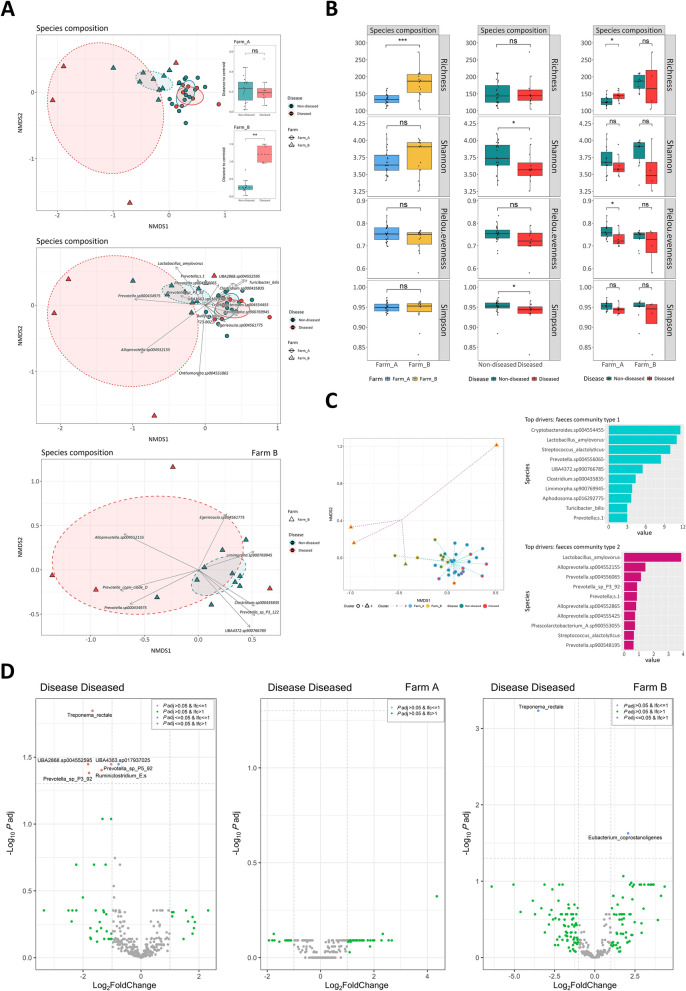
**Analysis of microbiota species composition in pre-SD samples**. **A** Beta diversity. NMDS ordination using Bray–Curtis distances at species composition. Boxplots indicate the distance to the centroid of each sample per disease variable according to the farm. ns *P* > 0.05, *P ≤ 0.05, **P ≤ 0.01, ***P ≤ 0.001 (Wilcoxon test or ANOVA and Tukey tests, with Holm adjustment). Ellipses represent covariance for each disease status according to the farm. The 30 species with the highest mean abundance and significant BH-adjusted *P*-value (by "envfit" model) are represented by arrows which pinpoint the sample ordination. The length of the arrow represents the strength of influence on the ordination (proportional to the *R*^*2*^ statistic by the "envfit" model). **B** Alpha diversity. Results of richness, Shannon, Pielou and Simpson indexes at species composition level by farm and disease status. ns *P* > 0.05, *P ≤ 0.05, **P ≤ 0.01, ***P ≤ 0.001, ****P ≤ 0.0001 (Wilcoxon test or ANOVA and Tukey tests, with Holm adjustment). **C** NMDS ordination of samples of each disease and farm within each DMM cluster. Contribution of the main species of the core microbiota to each cluster. **D** Species significantly increased or decreased (*P* < 0.05, log_2_FC > 1, BH-adjusted *P*-value) in the diseased pigs compared with non-diseased.

Further analyses to disclose differences in the microbiota background between SD diseased and non-diseased pigs were focused on samples collected before SD onset. The taxonomic analyses evidenced that sample ordination was influenced by disease variable, as indicated by the PERMANOVA results (*P* < 0.05) and differences in their variance (Levene test *P* < 0.05) (Figure 1A and Additional file 6). Pigs that later became diseased showed significantly lower species diversity before disease, as indicated by Shannon and Simpson indexes (*P* < 0.05) (Figure 1B and Additional file 4). In addition, these animals had a significantly lower abundance of *Treponema rectale*, *Prevotella* sp. P5-92, *Prevotella* sp. P3–92, *Ruminiclostridium* E, UBA2868 sp. 004552595 and UBA4363 sp. 017937025 (log_2_FC = −1.68, −0.77, −1.80, −1.36, −1.83, −1.02, *P* < 0.05) (Figure 1D and Additional file 8). Again, we observed significant differences in taxonomic and functions richness indexes between farms (*P* < 0.0001) (Figure 1B, Additional files 4, 5 and 9) and a strong influence of the farm variable on sample ordination (*P* < 0.001) (Figure 1A, Additional files 6, 7 and 9). Accordingly, samples from each farm were analysed separately, revealing significant differences on farm A, where pre-SD samples from diseased pigs showed higher species and function richness but lower species evenness (*P* < 0.05) (Figure 1B, Additional files 4, 5 and 9). On farm B, although no significant differences were observed in alpha diversity indexes (Figure 1B), sample clustering was influenced by the disease variable (*P* < 0.05), with significant taxonomic differences between diseased and non-diseased pigs (distance to centroid: *P* < 0.01; PERMANOVA: *P* < 0.05) (Figure 1A and Additional file 6). The ordination of diseased pigs was associated with specific taxa, including *Alloprevotella* sp. 004552155, *Prevotella copri* clade D and *Egerieousia* sp. 004561775 (Figure 1A). In addition, they exhibited a significant decrease in *Treponema rectale* (log_2_FC = −3.52; *P* < 0.001) and an increase in *Eubacterium coprostanoligenes* (log_2_FC = 2.07; *P* < 0.05) before disease (Figure 1D and Additional file 8).

To further investigate potential differences in microbial community structure among the monitored pigs, DMM models were applied. This analysis identified two distinct clusters, none of which were associated with the variables under study (disease, farm or sampling) (Fig 1C).

### Microbiota alterations in clinical disease

**Figure 2:**
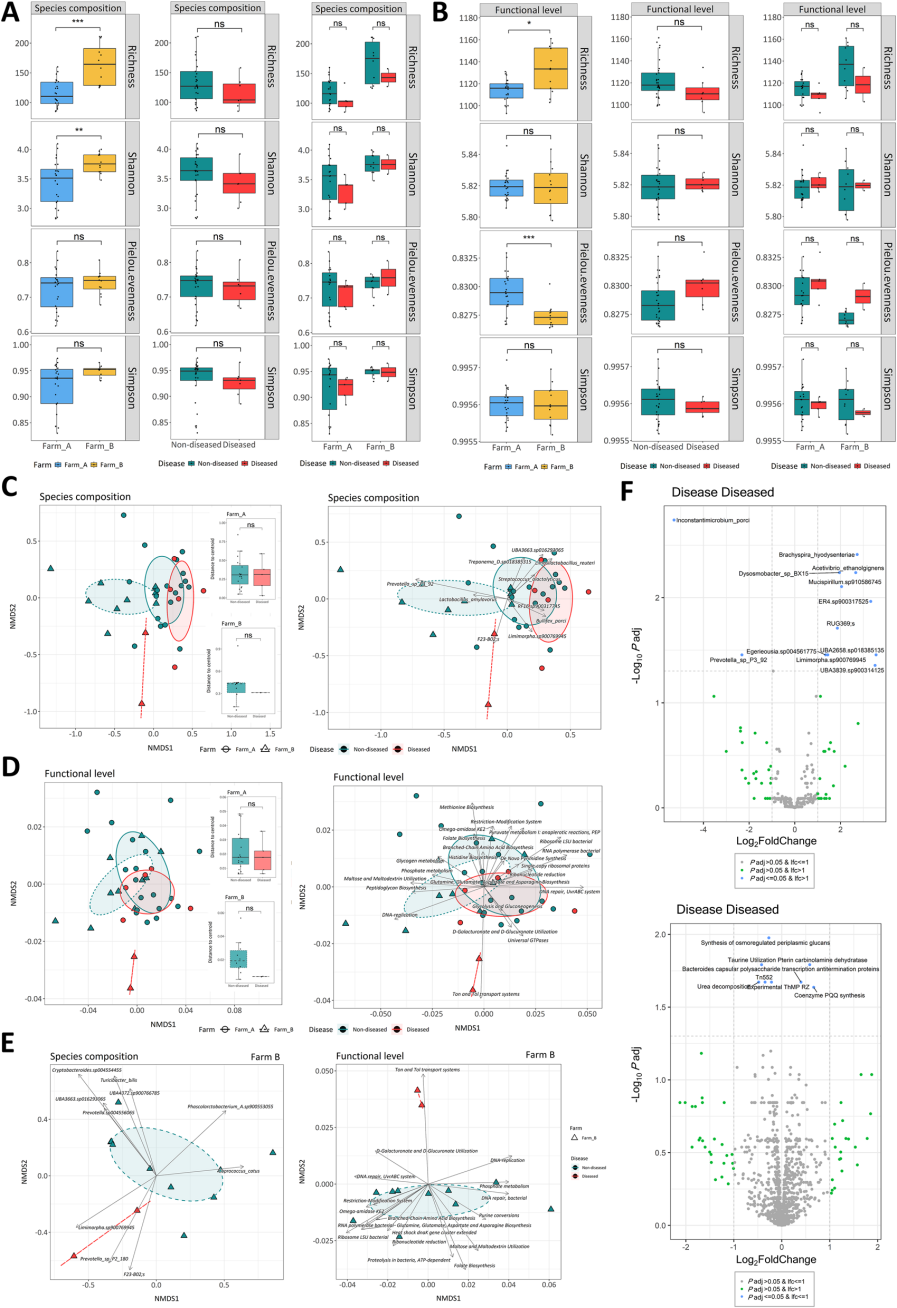
**Analysis of microbiota taxonomic and functional level in clinical-SD samples**. **A** Alpha diversity. Results of richness, Shannon, Pielou and Simpson indexes at species composition and B at functional level by farm and disease status. ns ***P*** > 0.05, *P ≤ 0.05, **P ≤ 0.01, ***P ≤ 0.001, ****P ≤ 0.0001 (Wilcoxon test or ANOVA and Tukey tests, with Holm adjustment). **C** Beta diversity. NMDS ordination using Bray–Curtis distances at species composition, **D** at functional level and **E** at species and functional level on farm B. Boxplots indicate the distance to the centroid of each sample per disease variable according to the farm. ns *P* > 0.05, *P ≤ 0.05, **P ≤ 0.01, ***P ≤ 0.001 (Wilcoxon test or ANOVA and Tukey tests, with Holm adjustment). Ellipses represent covariance for each disease status according to the farm. The 30 species and functions with the highest mean abundance and significant BH-adjusted *P*-value (by 'envfit' model) are represented by arrows which pinpoint the sample ordination. The length of the arrow represents the strength of influence on the ordination (proportional to the *R*^*2*^ statistic by the 'envfit' model). **F** Species and functions significantly increased or decreased (*P* < 0.05; BH-adjusted *P*-value) in the diseased pigs compared to non-diseased.

We further explored the changes of the microbiota during SD outbreak using confirmed clinical-SD samples from diseased pigs and samples collected at the same time point from non-diseased counterparts. Beta diversity analyses revealed that sample ordination was significantly influenced by the disease (*P* < 0.05) (Figures 2C, D and Additional files 6 and 7) while it did not alter significantly alpha diversity indexes (Figure 2A, B and Additional files 4 and 5). The same analyses also confirmed the impact of farm and sampling in the ordination of samples and the interaction between disease and sampling time point variables in species composition (Additional file 6). In addition, differences in species abundance were observed. *Brachyspira hyodysenteriae*, *Dysosmobacter* sp. BX15, *Acetivibrio ethanolgignens*, *Mucispirillum* sp. 910586745, ER4 sp. 900317525, RUG369, *Limimorpha* sp. 900769945, *Egerieousia* sp. 004561775, UBA2658 sp. 018385135 and UBA3839 sp. 900314125 showed a significant increase in their relative abundance (log_2_FC = 2.73, 1.95, 2.66, 2.04, 3.32, 1.86, 1.44, 1.35, 3.55, 3.51, *P* < 0.05), while *Inconstantimicrobium porci* and *Prevotella* sp. P3–92 decreased in abundance in diseased pigs during clinical SD compared with faeces from non-diseased pigs (log_2_FC = −5.29; −2.32; *P* < 0.05) (Figure 2F and Additional file 8).

Besides, we observed changes in functional abundances during clinical disease. Specifically, functions such as pterin carbinolamine dehydratase, *Bacteroides* capsular polysaccharide transcription antitermination proteins and coenzyme PQQ synthesis were increased (log_2_FC = 0.58, 0.40, 0.67, *P* < 0.05). In contrast, functions such as synthesis of osmoregulated periplasmic glucan, taurine utilization, urea decomposition, experimental ThMP RZ and Tn552 were decreased (log_2_FC = −0.27; −0.42; −0.48; −0.21; −0.35; *P* < 0.05) (Figure 2F and Additional file 8).

**Figure 3:**
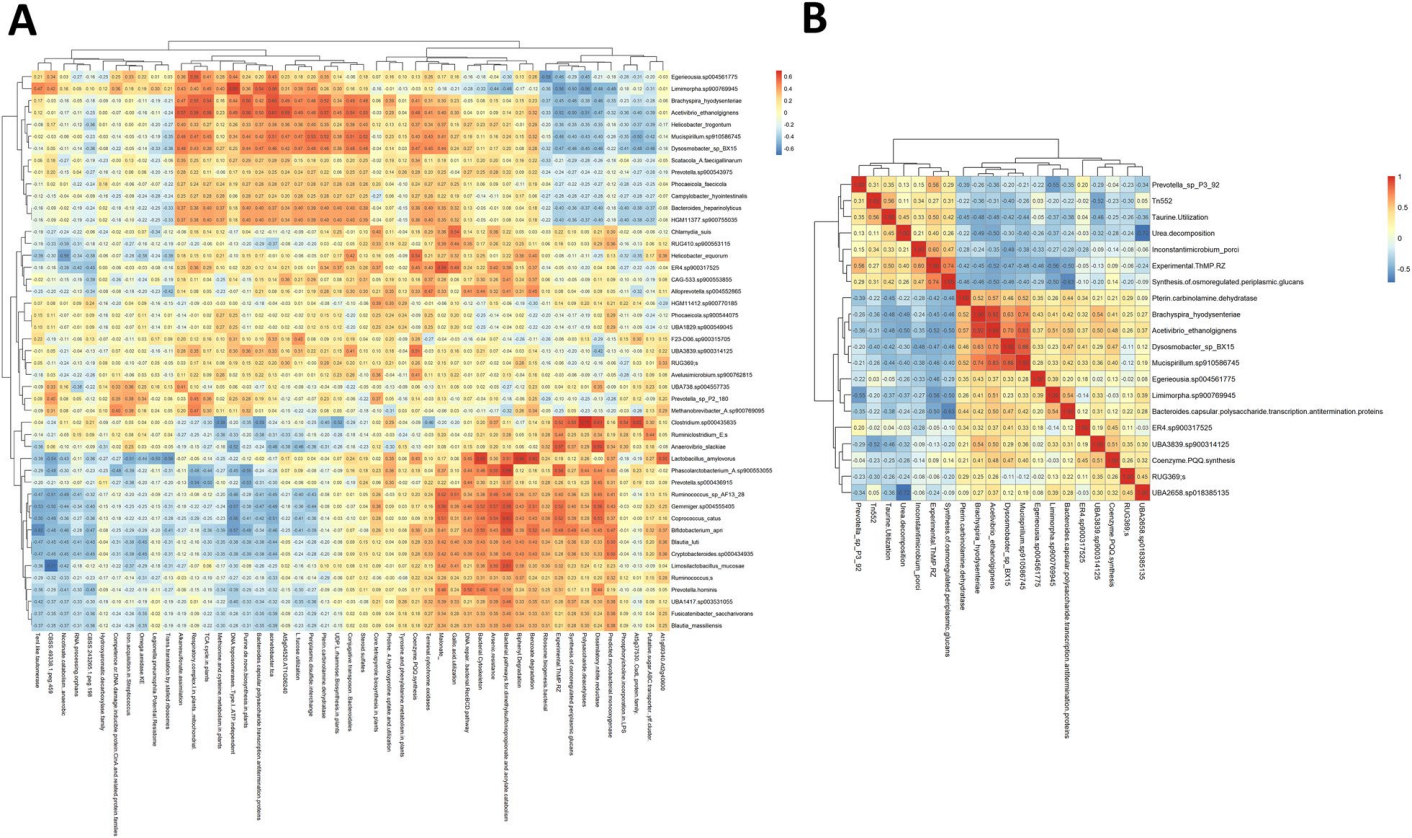
**Association analysis between microbial species and functions in clinical-SD samples**. **A** Heatmap showing the correlations between microbial key species and key functions associated with groups in disease variable, as identified by PLS-DA. **B** Heatmap displaying the correlation matrix between species and functions that showed significant abundance changes in the diseased pigs, as identified by LinDa. Similarities between species or functions are represented by a dendrogram.

**Figure 4:**
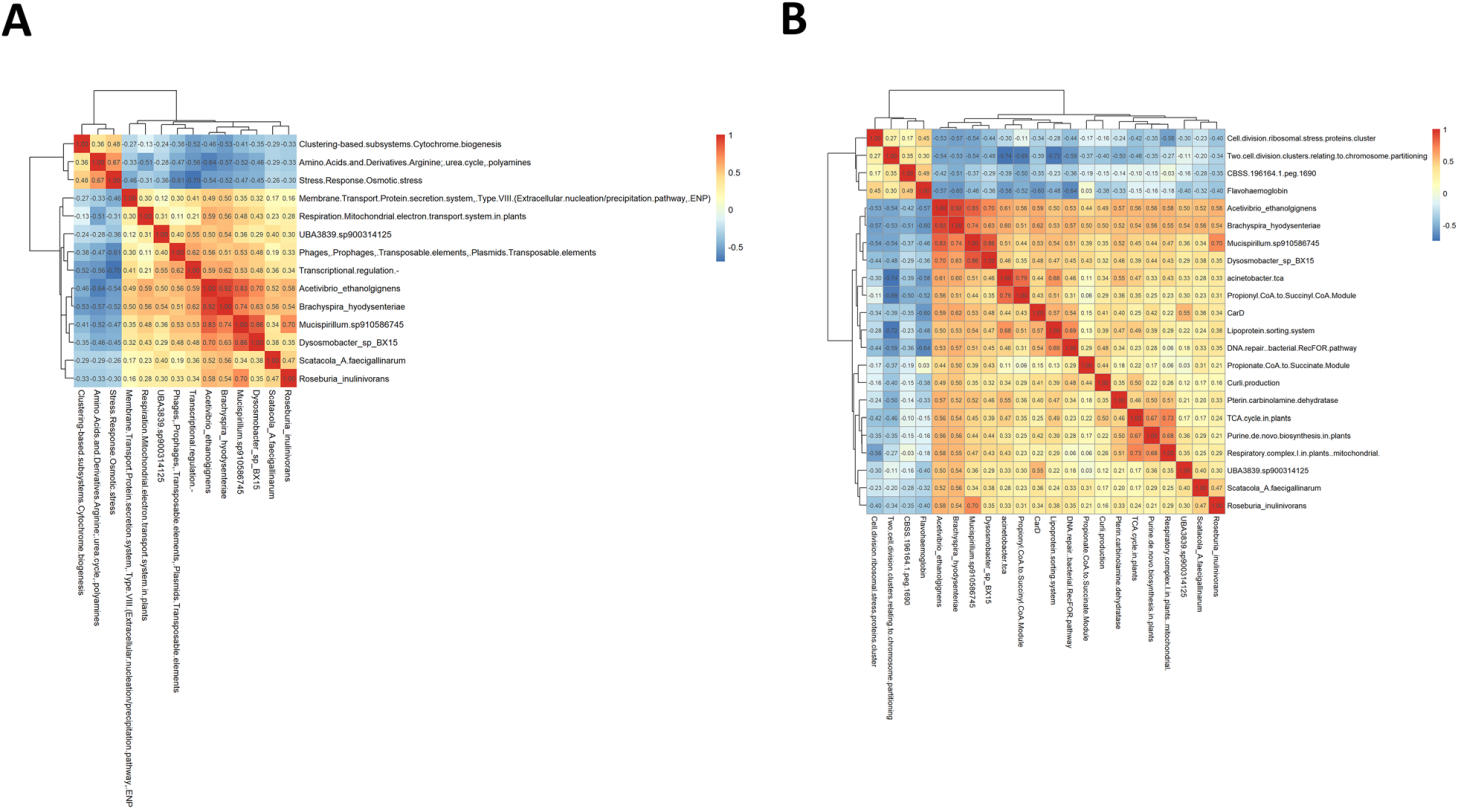
**Analysis of *B. hyodysenteriae*** **associations with microbial species and functions in clinical-SD samples**. **A** Heatmap showing significant correlations (rho > 0.5 or < −0.5 and *P* ≤ 0.05) between *B. hyodysenteriae* and microbial species, functional SUPER-FOCUS levels 1–2 and **B** SUPER-FOCUS level 3. Similarities between species or functions are represented by a dendrogram.

PLS-DA analysis of taxonomic and functional data by disease variable confirmed the results evidenced by differential abundance analysis (Additional file 10). This result allowed us to select species and functions of interest that were included in a correlation analysis (Figure 3A). Species were split into three clusters on the basis of their functionality, one of which included bacteria associated with the diseased pigs. This cluster exhibited negative correlations with functions such as, experimental ThMP RZ or dissimilatory nitrite reductase, which were positively correlated with the most representative bacteria in the non-diseased group. In addition, we observed that this cluster correlated positively with functions related to tricarboxylic acid cycle (TCA) and respiration (Figure 3A and Additional file 11). Further analysis of the correlations between all functions and the key species identified by PLS-DA revealed that those associated with the diseased group of pigs exhibited a similar functional profile during clinical SD, while species associated with non-diseased group were allocated in a different cluster (Additional file 12). Besides, a positive correlation was observed between *B. hyodysenteriae*, *A. ethanolgignens*, *Mucispirillum* sp. 910586745 and *Dysosmobacter* sp. BX15, as well as a negative correlation among *Prevotella* sp. P3–92, *Inconstantimicrobium porci* and these species, especially *Limimorpha* sp. 900769945 (Figure 3B and Additional file 11). Focusing on the significant correlations for *B. hyodysenteriae*, we observed a positive correlation with the function pterin carbinolamine dehydratase, which was elevated in diseased pigs during clinical disease, as well as with the species *Roseburia inulinivorans*. *Acetivibrio ethanolgignens* exhibited a similar profile showing positive correlations with functions such as propionyl CoA to succinyl CoA Module, propionate CoA to succinate module, curli production or transposable elements, and negative correlations with cytochrome biogenesis, arginine, urea cycle, polyamines, flavohaemoglobin and stress response (Figure 4).

When each farm was analysed independently, we observed that the influence of disease on the ordination was only maintained on farm B, where it was associated with three bacterial species: *Limimorpha* sp. 900769945, *Prevotella* sp. P2–180 and F23–B02 (Fig 2E).

### Re-establishment of the microbiota after swine dysentery recovery

Despite the differences observed in the microbiota composition between diseased and non-diseased pigs at the clinical SD, no significant differences in alpha or beta diversity were detected between them, either in the sampling immediately performed at post-infection (post-SD) or a13t the final sampling of the study (sampling 4) (Additional files 4, 5, 6, 7, and 14). At the post-SD sampling, a trend towards lower microbiota diversity and evenness (Shannon and Pielou indexes) was observed in recovered pigs, with significant differences in samples collected on farm A (*P* < 0.05) (Additional files 4 and 13 A). Despite the negligible differences in taxa, functional differences were observed, with diseased pigs exhibiting higher functional richness index (*P* < 0.05) (Additional files 5 and 14 A). This pattern was also observed in pigs from farm A. This difference, however, was no longer observed at the final sampling (Additional files 4, 5, 13 B and 14 B). Likewise, no significant changes in species abundance were detected, except for an increase in CAG-533 sp. 000434495 (log_2_FC = 1.79; *P* < 0.05), which was accompanied by an increase in the denitrification function (log_2_FC = 0.22; *P* < 0.05) at the final sampling in diseased pigs from farm B (Additional files 8, 13 F and 14 F).

Similarly, no significant differences were found between pre-SD and post-SD samples in diseased pigs (Additional files 4, 5, 6, 7, 15 and 16), except for a significant increase after disease in *Treponema D* sp. 018385315 (log_2_FC = 1.33; *P* < 0.01) and total richness index value for functional data (*P* < 0.05) (Additional files 15 F and 16 B). However, when analysing the farms separately, on farm A, PERMANOVA analysis revealed significant differences between pre-SD and post-SD samples in diseased pigs, characterized by an increase in *Treponema D* sp. 018385315 (log_2_FC = 1.21; *P* < 0.05), UMGS1470 sp. 900552105 (log_2_FC = 3.98; *P* < 0.05) and a decrease in *Alloprevotella* sp. 004552865 (log_2_FC = −2.59; *P* < 0.05) in the post-SD samples (Additional files 8 and 15 F). A larger dispersion in the ordination of pre-SD samples from diseased pigs was observed on farm B compared with post-SD samples (distance to centroid, *P* < 0.05). This dispersion was not observed in non-diseased pigs pre-SD and post-SD samples (Additional files 15 C and D). Furthermore, an increase in the abundance of UBA3663 sp. 016293065 and a decrease in *Anaerovibrio slackiae* were detected (log_2_FC = 9.58; −1.61; *P* < 0.01, respectively) in these samples from non-diseased pigs (Additional files 8 and 15 E). Differences in pre-SD samples and post-SD samples in non-diseased group from this farm were limited to an increase in functional diversity by Shannon and Simpson indexes (Additional files 5 and 16 A).

## Discussion

Although current knowledge on SD remains limited, since decades ago, the host microbiota has been postulated as part of the factors influencing the *B. hyodysenteriae* infection outcome [20, 47]. Several studies have addressed this topic under experimental conditions [21–23], where environmental and biological variables are tightly controlled but which may do not comprise all factors involved under field conditions. In contrast, studying the disease in natural settings may be influence by non-controlled factors, such as co-infections, husbandry, nutrition or production stage, among others, which can complicate data interpretation. Altogether it provides information complementary to the data gathered by controlled challenges so far and may provide novel data about the role of the microbiota in disease susceptibility or resilience. The identification of potential confounding factors are important in the analysis the microbiota under field conditions to avoid biases. Here we observed that both, sampling (animal age) and farm, impacted the structure and composition of the gut microbiota, such as previous studies [8, 48] and both factors were considered in the analyses performed.

The microbiota assembly in early life seem to have a crucial role in host health in later stages [49]. The microbial diversity and composition may change gut permissiveness to pathogen colonisation [50]. Numerous studies have shown that less complex and stable microbial profiles, characterized by reduced bacterial diversity and evenness, can negatively impact the host health [51, 52]. In line with these results, we observed that pigs that ended in a clinical infection, first had a trend in lower diversity at the beginning of the study (around weaning age) which was confirmed statistically in the sampling performed before the disease onset. Another field study evidenced a higher richness in the microbiota of *Salmonella*-free pigs compared with animals that became infected [53]. Apart from global richness, certain taxa could also exert a competitive exclusion against pathogens [54]. Swine dysentery susceptible pigs exhibited a lower abundance of *Treponema rectale*, *Prevotella* sp. P5–92, *Prevotella* sp. P3–92, *Ruminiclostridium* E, UBA2868 sp. 004552595 and UBA4363 sp. 017937025 in pre-SD samples. The loss of bacterial genera such as *Treponema*, which plays a key role in the degradation of lignin and cellulose [55], or *Prevotella*, which is involved in the metabolism of complex dietary carbohydrates and the production of short-chain fatty acids, important for the development and maintenance of immune defence [56], may render these animals more susceptible to *B. hyodysenteriae* infection and disease.

The analysis of data on each farm revealed that on farm B, SD susceptible pigs showed lower abundance of *Roseburia* sp. 499 and *Treponema rectale* and an increased abundance in *Eubacterium coprostanoligenes*. The last has been associated with the expression of MUC2 [57], a mucin involved in intestinal mucosa protection that is upregulated in SD [15, 58]. However, a higher abundance of *Eubacterium* spp. has also been reported in samples collected prior to the onset of mucohaemorrhagic diarrhoea [22]. These findings, together with our observations, may suggest a role in facilitating *B. hyodysenteriae* colonization, although further investigation is required.

Microbiota changes in pigs with mucohaemorrhagic diarrhoea evidenced changes in species composition and functional profiles. However, consistent with the findings of Fodor et al. [23], we did not detect significant differences in alpha diversity indexes, although a decreased tendency in richness, Shannon, Pielou and Simpson indexes was observed [21, 59]. Pigs in acute SD not only exhibited an increase in *B. hyodysenteriae*, but a concurrent increase in other correlated bacteria, such as *Dysosmobacter* sp. BX15, *Muscispirillum* sp. 910586745 and *A. ethanolgignens*. The increase in *A. ethanolgignens* in association with SD has previously been reported in experimental infections [23, 60], reinforcing its potential role in the pathogenesis of the disease. From a functional perspective, the increase in Gram-negative bacteria observed in SD affected pigs was evidenced by an enhanced transcription of *Bacteroides* capsular polysaccharides, which are related to the cell wall structure of these bacteria. Conversely, a reduction of urea decomposition and taurine utilization observed may exacerbate intestinal epithelial damage and impair barrier function [61, 62], thereby contributing to exudative diarrhoea and increased solute concentration in the intestine, while reducing the synthesis of osmoregulated periplasmic glucans [63], all factors that could contribute to the clinical outcome of the dysentery.

Dysbiosis, epithelial damage and inflammation contribute to a more oxidative intestinal environment [64]. Accordingly, increased bacteria in the diseased pigs exhibited inverse correlations with processes such as dissimilatory nitrite reduction, linked to nitrogen metabolism in anaerobic environments including the colon [65]. In addition, negative associations were established with thiamine monophosphate metabolism (experimental ThMP RZ), contributing to the intestinal dysfunction by altering energy-related processes and exacerbating oxidative stress and intestinal inflammation [66]. In contrast, we observed a positive correlation with L-fucose utilization, which is overproduced during SD as a result of mucin glycosylation [67]. This increase in L-fucose utilization is likely driven by microbial displacement that causes the degradation of the glycans of the mucous layer, releasing sugars such as fucose, which favour the growth of pathogenic species [64, 68]. As a result, the intestinal metabolism becomes more oxidative, as reflected by increased TCA activity, favouring the proliferation of species with more flexible metabolic capabilities [69]. Contradictory results were observed between the disease pigs and bacteria from the genus *Roseburia*. The positive correlation between *B. hyodysenteriae* and *Roseburia inulinivorans* is noteworthy. *Roseburia* is a commensal member of the porcine intestinal microbiota that produces butyrate, a short-chain fatty acid with multiple beneficial effects on host health [70]. However, previous studies on SD have also reported an increase in this bacterium [23], which may suggest that *R. inulinivorans* benefits from the altered metabolic or mucin-rich environment, characteristic of this disease, owing to its capacity to utilize fucose present in the large quantities of mucins released [71].

As mentioned above, in this study *B. hyodysenteriae* relative abundance showed positive correlations with other anaerobic and facultative anaerobic bacteria, as well as functions such as propionyl CoA to succinyl CoA module and propionate CoA to succinate module. These findings suggest that these bacteria may collaborate in the pathogenesis of *B. hyodysenteriae* infection by increasing succinic acid levels in the intestinal lumen, potentially contributing to the development of colitis and altering polymorphonuclear cell function [72]. Similarly, the combined effect of decreased flavohaemoglobin levels and increased pterin carbinolamine dehydratase, an enzyme involved in tetrahydrobiopterin regeneration, may led to elevated nitric oxide production through anaerobic respiration, which have been associated with tissue damage in diseases such as inflammatory bowel disease [73–75].

Compared with challenge studies that are usually constrained in the monitored time-period, field studies allow us to monitor pigs after the recovery of the disease for a longer period of time. To our knowledge, this is the first study that analyses the effects of the SD infection in the microbiota composition after treatment and recovery of the diseased animals. In the sampling performed after clinical disease, we observed a trend towards reduction in species diversity and evenness. The result may be associated either to the infection, to the treatment with lincomycin or to their combination. Indeed, previous studies on the effects of antibiotics on gut microbiota composition have reported a loss of diversity and changes in abundance of specific taxa [76, 77]. The loss of commensal microbiota may lead to the expansion of adaptive functions in response to the new environment, including the activation of alternative metabolic pathways, resistance mechanisms and stress response systems [76, 77]. This functional shift could explain the increased microbial functionality observed in these animals. However, the porcine gut microbiota exhibits considerable resilience to long-term alterations following antibiotic-induced perturbations [78] and the resistance to lincosamides are relatively frequent among pig gut commensals [79]. In recovered pigs, we observed an increase in denitrification, indicating a return to anaerobic conditions in the colon [75]. Compared with pre-SD samples, the microbiota after recovery appeared to have a more mature profile, with an increase in bacteria such as *Treponema* spp. and higher uniformity, which may have been influenced by the antibiotic treatment [80]. Consistent with this finding, after the onset of mucohaemorrhagic diarrhoea, we did not observe significant differences in the microbiota among recovered pigs compared with controls that would suggest lasting consequences of the disease, at least under the treatment conditions applied in this study.

This first field study monitoring microbiota changes in natural SD reveals that, as observed with other enteric pathogens, a less diverse and less uniform gut microbiota, along with a reduced abundance of certain taxa considered beneficial to the host, may increase susceptibility to SD disease. Once infection is established and mucohaemorrhagic diarrhoea has developed, major taxonomic and functional microbial shifts occur as adaptations to the inflamed and redox altered intestinal environment, contributing to the clinical outcome of the dysentery. Despite these alterations, our findings indicate that *B. hyodysenteriae* infection does not induce long-term changes in the intestinal microbiota, at least under conditions where antibiotic therapy is administered.

## Supplementary Information

**Additional file 1**. Sheet 1. Feed formulation data, including the formula, period of administration and presentation. Sheet 2. Metadata from 102 samples selected to perform the study.

**Additional file 2. Analysis of microbiota species composition in sampling 1**. **A** Alpha diversity. Results of richness, Shannon, Pielou and Simpson indexes by farm and disease status. ns *P* > 0.05, *P ≤ 0.05, **P ≤ 0.01, ***P ≤ 0.001, ****P ≤ 0.0001 (Wilcoxon test or ANOVA and Tukey tests, with Holm adjustment). **B** Beta diversity. NMDS ordination using Bray-Curtis distances. Boxplots indicate the distance to the centroid of each sample per disease variable according to the farm. ns *P* > 0.05, *P ≤ 0.05, **P ≤ 0.01, ***P ≤ 0.001 (Wilcoxon test or ANOVA and Tukey tests, with Holm adjustment). Ellipses represent covariance for each disease status according to the farm. The 30 species with the highest mean abundance and significant BH-adjusted *P*-value (by “envﬁt” model) are represented by arrows which pinpoint the sample ordination. The length of the arrow represents the strength of inﬂuence on the ordination (proportional to the R2 statistic by the “envﬁt” model). **C** Species significantly increased or decreased (*P* < 0.05, log_2_FC > 1; BH-adjusted *P*-value) in the diseased pigs compared to non-diseased.

**Additional file 3. Analysis of microbiota functional level in sampling 1**. **A** Alpha diversity. Results of richness, Shannon, Pielou and Simpson indexes by farm and disease status. ns *P* > 0.05, *P ≤ 0.05, **P ≤ 0.01, ***P ≤ 0.001, ****P ≤ 0.0001 (Wilcoxon test or ANOVA and Tukey tests, with Holm adjustment). **B** Beta diversity. NMDS ordination using Bray-Curtis distances. Boxplots indicate the distance to the centroid of each sample per disease variable according to the farm. ns *P* > 0.05, *P ≤ 0.05, **P ≤ 0.01, ***P ≤ 0.001 (Wilcoxon test or ANOVA and Tukey tests, with Holm adjustment). Ellipses represent covariance for each disease status according to the farm. The 30 functions with the highest mean abundance and significant BH-adjusted *P*-value (by “envﬁt” model) are represented by arrows which pinpoint the sample ordination. The length of the arrow represents the strength of inﬂuence on the ordination (proportional to the *R*^*2*^ statistic by the “envﬁt” model). **C** Functions significantly increased or decreased (*P* < 0.05, log_2_FC > 1; BH-adjusted *P*-value) in the diseased pigs compared to non-diseased.

**Additional file 4. Taxonomic alpha diversity indexes.**

**Additional file 5. Functional alpha diversity indexes.**

** Additional file 6. Taxonomic beta diversity analyses**. Influence of different factors on the ordination of samples and results of the multivariate permutational analysis of variance in the Bray-Curtis distances of the ordination.

**Additional file 7. Functional beta diversity analyses**. Influence of different factors on the ordination of samples and results of the multivariate permutational analysis of variance in the Bray-Curtis distances of the ordination.

**Additional file 8. Results of microbiota linear models for differential abundance species and functions.**

**Additional file 9. Analysis of microbiota functional level in pre-SD samples.** **A** Alpha diversity. Results of richness, Shannon, Pielou and Simpson indexes by farm and disease status. ns *P* > 0.05, *P ≤ 0.05, **P ≤ 0.01, ***P ≤ 0.001, ****P ≤ 0.0001 (Wilcoxon test or ANOVA and Tukey tests, with Holm adjustment). **B** Beta diversity. NMDS ordination using Bray-Curtis distances. Boxplots indicate the distance to the centroid of each sample per disease variable according to the farm. ns *P* > 0.05, *P ≤ 0.05, **P ≤ 0.01, ***P ≤ 0.001 (Wilcoxon test or ANOVA and Tukey tests, with Holm adjustment). Ellipses represent covariance for each disease status according to the farm. The 30 functions with the highest mean abundance and significant BH-adjusted *P*-value (by “envﬁt” model) are represented by arrows which pinpoint the sample ordination. The length of the arrow represents the strength of inﬂuence on the ordination (proportional to the *R*^*2*^ statistic by the “envﬁt” model). **C** Functions significantly increased or decreased (*P* < 0.05, log_2_FC > 1; BH-adjusted *P*-value) in the diseased pigs compared to non-diseased.

**Additional file 10. PLS-DA analysis of differences in microbial species composition and functional level between diseased and non-diseased groups.**

**Additional file 11. Heatmap showing the correlation matrix between microbial key species and key functions associated with groups in disease variable, as identified by PLS-DA analysis in clinical-SD samples.** Similarities between species or functions are represented by a dendrogram.

**Additional file 12. Heatmap showing significant correlations** (rho > 0.5 or < − 0.5 and *P* ≤ 0.05) between microbial key species associated with groups in disease variable, as identified by PLS-DA analysis and all functions in clinical-SD samples. Similarities between species or functions are represented by a dendrogram.

**Additional file 13. Analysis of microbiota species composition in post-SD and sampling 4 samples**. **A** Alpha diversity. Results of richness, Shannon, Pielou and Simpson indexes by farm and disease status in post-SD samples and **B** in samples from sampling 4. ns *P* > 0.05, *P ≤ 0.05, **P ≤ 0.01, ***P ≤ 0.001, ****P ≤ 0.0001 (Wilcoxon test or ANOVA and Tukey tests, with Holm adjustment). **C** Beta diversity. NMDS ordination using Bray-Curtis distances in post-SD samples and **D** in samples from sampling 4. Boxplots indicate the distance to the centroid of each sample per disease variable according to the farm. ns *P* > 0.05, *P ≤ 0.05, **P ≤ 0.01, ***P ≤ 0.001 (Wilcoxon test or ANOVA and Tukey tests, with Holm adjustment). Ellipses represent covariance for each disease status according to the farm. The 30 species with the highest mean abundance and significant BH-adjusted *P*-value (by “envﬁt” model) are represented by arrows which pinpoint the sample ordination. The length of the arrow represents the strength of inﬂuence on the ordination (proportional to the *R*^*2*^ statistic by the “envﬁt” model). **E** Species significantly increased or decreased (*P* < 0.05, log_2_FC > 1; BH-adjusted *P*-value) in the diseased compared to non-diseased pigs in post-SD samples and **F** in samples from sampling 4.

**Additional file 14. Analysis of microbiota functional level in post-SD and sampling 4 samples**. **A** Alpha diversity. Results of richness, Shannon, Pielou and Simpson indexes by farm and disease status in post-SD samples and **B** in samples from sampling 4. ns *P* > 0.05, *P ≤ 0.05, **P ≤ 0.01, ***P ≤ 0.001, ****P ≤ 0.0001 (Wilcoxon test or ANOVA and Tukey tests, with Holm adjustment). **C** Beta diversity. NMDS ordination using Bray-Curtis distances in post-SD samples and **D** in samples from sampling 4. Boxplots indicate the distance to the centroid of each sample per disease variable according to the farm. ns *P* > 0.05, * *P* ≤ 0.05, ** *P* ≤ 0.01, *** *P* ≤ 0.001 (Wilcoxon test or ANOVA and Tukey tests, with Holm adjustment). Ellipses represent covariance for each disease status according to the farm. The 30 functions with the highest mean abundance and significant BH-adjusted *P*-value (by “envﬁt” model) are represented by arrows which pinpoint the sample ordination. The length of the arrow represents the strength of inﬂuence on the ordination (proportional to the *R*^*2*^ statistic by the “envﬁt” model). **E** Functions significantly increased or decreased (*P* < 0.05, log_2_FC > 1; BH-adjusted *P*-value) in the diseased compared to non-diseased pigs in post-SD samples and **F** in samples from sampling 4.

**Additional file 15. Analysis of microbiota species composition in non-diseased and diseased pigs: comparison between pre-SD and post-SD samples**. **A** Alpha diversity. Results of richness, Shannon, Pielou and Simpson indexes by farm and dysentery status in non-diseased and **B** in diseased pigs. ns *P* > 0.05, *P ≤ 0.05, **P ≤ 0.01, ***P ≤ 0.001, ****P ≤ 0.0001 (Wilcoxon test or ANOVA and Tukey tests, with Holm adjustment). **C** Beta diversity. NMDS ordination using Bray-Curtis distances in non-diseased and **D** diseased pigs. Boxplots indicate the distance to the centroid of each sample per dysentery variable according to the farm. ns *P* > 0.05, *P ≤ 0.05, **P ≤ 0.01, ***P ≤ 0.001 (Wilcoxon test or ANOVA and Tukey tests, with Holm adjustment). Ellipses represent covariance for each dysentery status according to the farm. The 30 species with the highest mean abundance and significant BH-adjusted *P*-value (by “envﬁt” model) are represented by arrows which pinpoint the sample ordination. The length of the arrow represents the strength of inﬂuence on the ordination (proportional to the *R*^*2*^ statistic by the “envﬁt” model). **E** Species significantly increased or decreased (*P* < 0.05, log_2_FC > 1; BH-adjusted *P*-value) in the post-SD compared to pre-SD samples in non-diseased and **F** diseased pigs.

**Additional file 16. Analysis of microbiota functional level in non-diseased and diseased pigs: comparison between pre-SD and post-SD samples**. **A** Alpha diversity. Results of richness, Shannon, Pielou and Simpson indexes by farm and dysentery status in non-diseased and **B** in diseased pigs. ns *P* > 0.05, *P ≤ 0.05, **P ≤ 0.01, ***P ≤ 0.001, ****P ≤ 0.0001 (Wilcoxon test or ANOVA and Tukey tests, with Holm adjustment). **C** Beta diversity. NMDS ordination using Bray-Curtis distances in non-diseased and **D** diseased pigs. Boxplots indicate the distance to the centroid of each sample per dysentery variable according to the farm. ns *P* > 0.05, *P ≤ 0.05, **P ≤ 0.01, ***P ≤ 0.001 (Wilcoxon test or ANOVA and Tukey tests, with Holm adjustment). Ellipses represent covariance for each dysentery status according to the farm. The 30 functions with the highest mean abundance and significant BH-adjusted *P*-value (by “envﬁt” model) are represented by arrows which pinpoint the sample ordination. The length of the arrow represents the strength of inﬂuence on the ordination (proportional to the *R*^*2*^ statistic by the “envﬁt” model). **E** Functions significantly increased or decreased (*P* < 0.05, log_2_FC > 1; BH-adjusted *P*-value) in the post-SD compared to pre-SD samples in non-diseased and **F** diseased pigs.

## Data Availability

The metagenomic sequences and its associated metadata have been submitted to NCBI (SRA) and are available under BioProject accession PRJNA1218713: [https://www.ncbi.nlm.nih.gov/sra/PRJNA1218713] .

## Abbreviations

ANOVA analysis of variance BH: Benjamini–Hochberg
DMM: Dirichlet multinomial mixtures
FC: fold change
LinDA: linear models for differential abundance
NMDS: Non-metric Multidimensional Scaling
PERMANOVA: permutational multivariate analysis of variance
PLS-DA: Partial Least Squares Discriminant Analysis
SD: swine dysentery
SRA: Sequence Read Archive
TCA: tricarboxylic acid cycle
