# Supplementary material for: From predisposition to recovery: field evidence of interactions between the gut microbiota and Brachyspira hyodysenteriae infection
Source: Vet Res. 2026 Jan 30;57:25. doi: 10.1186/s13567-025-01646-1 (PMC12857038; doi:10.1186/s13567-025-01646-1)
Supplement: Supplementary file 4 — Additional file 4. Taxonomic alpha diversity indexes. [file 13567_2025_1646_MOESM4_ESM.docx]

**Additional file 4** Taxonomic alpha-diversity indexes.

| Factor | | Richness | Shannon | Pielou evenness | Simpson |
| --- | --- | --- | --- | --- | --- |
|  |  | *P*-value | *P*-value | *P*-value | *P*-value |
| Sampling 1 | | | | | |
| Farm A vs Farm B | | 0.0041 ** | 0.0649 | 0.8300 | 0.6000 |
| Diseased vs Non-diseased | | 0.4800 | 0.0869 | 0.3500 | 0.4500 |
|  | Farm A – Diseased vs Non-diseased | 0.3160 | 0.8438 | 0.3398 | 0.4320 |
|  | Farm B - Diseased vs Non-diseased | 0.6138 | 0.0912 | 0.9000 | 0.7300 |
| Pre-SD sampling | | | | | |
| Farm A vs Farm B | | 0.0003 *** | 0.3673 | 0.3400 | 0.7900 |
| Sampling 1 vs Sampling 2 | | 0.9100 | 0.1774 | 0.1300 | 0.0680 |
| Diseased vs Non-diseased | | 0.9700 | 0.0477 * | 0.0670 | 0.0450 * |
|  | Farm A - Diseased vs Non-diseased | 0.0268 * | 0.3250 | 0.0255 * | 0.0651 |
|  | Farm B - Diseased vs Non-diseased | 0.8172 | 0.3000 | 0.8400 | 0.4500 |
| Clinical SD sampling | | | | | |
| Farm A vs Farm B | | 0.0002 *** | 0.0079 ** | 0.3543 | 0.0720 |
| Sampling_1 vs Sampling_2 | | 0.0078 ** | 1.0000 | 0.1959 | 1.0000 |
| Sampling_1 vs Sampling_3 | | 0.0012 ** | 0.0060 ** | 0.6366 | 0.4200 |
| Sampling_2 vs Sampling_3 | | 0.0750 | 0.0035 ** | 0.0049 ** | 0.0090 ** |
| Diseased vs Non-diseased | | 0.1900 | 0.2100 | 0.7928 | 0.2700 |
|  | Farm A - Diseased vs Non-diseased | 0.2076 | 0.3950 | 0.6163 | 0.3700 |
|  | Farm B - Diseased vs Non-diseased | 0.3524 | 0.9107 | 0.4832 | 0.9647 |
| Post-SD sampling | | | | | |
| Farm A vs Farm B | | 0.0110 * | 0.0255 * | 0.2539 | 0.0063 ** |
| Sampling_2 vs Sampling_4 | | 0.5500 | 0.0679 | 0.0514 | 0.0850 |
| Diseased vs Non-diseased | | 0.4700 | 0.0521 | 0.0643 | 0.1300 |
|  | Farm A - Diseased vs Non-diseased | 0.7109 | 0.0495 * | 0.0307 * | 0.1200 |
|  | Farm B - Diseased vs Non-diseased | 0.4022 | 0.6856 | 0.8468 | 0.6400 |
| Sampling 4 | | | | | |
| Farm A vs Farm B | | 0.0675 | 0.0008 *** | 0.0046 ** | 0.0001 *** |
| Diseased vs Non-diseased | | 0.2010 | 0.2481 | 0.5595 | 0.5400 |
|  | Farm A - Diseased vs Non-diseased | 0.1612 | 0.3160 | 0.8456 | 0.6348 |
|  | Farm B - Diseased vs Non-diseased | 0.8317 | 0.4895 | 0.5246 | 0.4100 |
| Pre-SD and Post-SD Non-diseased pigs | | | | | |
| Farm A vs Farm B | | 5.80e-06 **** | 0.1098 | 0.3890 | 0.4500 |
| Sampling_1 vs Sampling_2 | | 0.9990 | 0.3536 | 0.1562 | 0.2500 |
| Sampling_1 vs Sampling_3 | | 0.3754 | 0.2014 | 0.7608 | 1.0000 |
| Sampling_1 vs Sampling_4 | | 0.1000 | 0.9784 | 0.9933 | 1.0000 |
| Sampling_2 vs Sampling_3 | | 0.3847 | 0.0379 * | 0.1895 | 1.0000 |
| Sampling_2 vs Sampling_4 | | 0.9969 | 0.6565 | 0.3062 | 1.0000 |
| Sampling_3 vs Sampling_4 | | 0.3680 | 0.1459 | 0.6851 | 1.0000 |
| Pre-SD vs Post-SD | | 0.8687 | 0.9203 | 0.8394 | 0.6100 |
|  | Farm A - Pre-SD vs Post-SD | 0.6053 | 0.9532 | 0.7442 | 0.9600 |
|  | Farm B - Pre-SD vs Post-SD | 0.2432 | 0.6978 | 0.2733 | 0.3381 |
| Pre-SD and Post-SD Diseased pigs | | | | | |
| Farm A vs Farm B | | 0.4681 | 0.1326 | 0.1199 | 0.0100 ** |
| Sampling_1 vs Sampling_2 | | 0.9986 | 0.9983 | 0.9992 | 1.0000 |
| Sampling_1 vs Sampling_3 | | 0.3941 | 0.0103 * | 0.0394 * | 1.0000 |
| Sampling_1 vs Sampling_4 | | 0.9973 | 0.9728 | 0.9732 | 1.0000 |
| Sampling_2 vs Sampling_3 | | 0.4091 | 0.0069 ** | 0.0289 * | 1.0000 |
| Sampling_2 vs Sampling_4 | | 0.9999 | 0.9213 | 0.9329 | 1.0000 |
| Sampling_3 vs Sampling_4 | | 0.4648 | 0.0189 * | 0.0681 | 1.0000 |
| Pre-SD vs Post-SD | | 0.5631 | 0.3171 | 0.3778 | 0.7000 |
|  | Farm A - Pre-SD vs Post-SD | 0.2741 | 0.1273 | 0.2012 | 0.4400 |
|  | Farm B - Pre-SD vs Post-SD | 0.9063 | 0.6515 | 0.7042 | 0.3529 |

* *P* ≤ 0.05, ** *P* ≤ 0.01, *** *P* ≤ 0.001, **** *P* ≤ 0.0001
