# Supplementary material for: From predisposition to recovery: field evidence of interactions between the gut microbiota and Brachyspira hyodysenteriae infection
Source: Vet Res. 2026 Jan 30;57:25. doi: 10.1186/s13567-025-01646-1 (PMC12857038; doi:10.1186/s13567-025-01646-1)
Supplement: Supplementary file 5 — Additional file 5. Functional alpha diversity indexes. [file 13567_2025_1646_MOESM5_ESM.docx]

**Additional file 5** Functional alpha-diversity indexes.

| Factor | | Richness | Shannon | Pielou evenness | Simpson |
| --- | --- | --- | --- | --- | --- |
|  |  | *P*-value | *P*-value | *P*-value | *P*-value |
| Sampling 1 | | | | | |
| Farm A vs Farm B | | 0.4100 | 0.6923 | 0.8300 | 0.8171 |
| Diseased vs Non-diseased | | 0.9300 | 0.9964 | 1.0000 | 0.7940 |
|  | Farm A - Diseased vs Non-diseased | 0.2000 | 0.1280 | 0.5811 | 0.1828 |
|  | Farm B - Diseased vs Non-diseased | 0.2200 | 0.1017 | 1.0000 | 0.1293 |
| Pre-SD sampling | | | | | |
| Farm A vs Farm B | | 0.0220 * | 0.6592 | 0.3400 | 0.7765 |
| Sampling 1 vs Sampling 2 | | 0.0150 * | 0.6719 | 0.0970 | 0.8911 |
| Diseased vs Non-diseased | | 0.3100 | 0.4411 | 1.0000 | 0.9397 |
|  | Farm A - Diseased vs Non-diseased | 0.0370 * | 0.3000 | 0.9631 | 0.2665 |
|  | Farm B - Diseased vs Non-diseased | 1.0000 | 0.3639 | 1.0000 | 0.3700 |
| Clinical SD sampling | | | | | |
| Farm A vs Farm B | | 0.0140 * | 0.8119 | 0.0006 *** | 0.9486 |
| Sampling_1 vs Sampling_2 | | 0.0053 ** | 0.1850 | 0.1379 | 0.6752 |
| Sampling_1 vs Sampling_3 | | 0.0024 *** | 0.0958 | 0.0865 | 0.2250 |
| Sampling_2 vs Sampling_3 | | 1.0000 | 0.9930 | 0.9990 | 0.6833 |
| Diseased vs Non-diseased | | 0.1000 | 0.6819 | 0.0526 | 0.4725 |
|  | Farm A - Diseased vs Non-diseased | 0.1331 | 0.6430 | 0.2109 | 0.7309 |
|  | Farm B - Diseased vs Non-diseased | 0.3085 | 0.9186 | 0.0610 | 0.4966 |
| Post-SD sampling | | | | | |
| Farm A vs Farm B | | 0.6533 | 0.8621 | 0.5237 | 0.4797 |
| Sampling_2 vs Sampling_4 | | 0.0174 * | 0.0070 ** | 0.8454 | 0.0167 * |
| Diseased vs Non-diseased | | 0.0074 ** | 0.3325 | 0.0537 | 0.6123 |
|  | Farm A - Diseased vs Non-diseased | 0.0387 * | 0.4608 | 0.1369 | 0.6869 |
|  | Farm B - Diseased vs Non-diseased | 0.1064 | 0.5708 | 0.2246 | 0.7302 |
| Sampling 4 | | | | | |
| Farm A vs Farm B | | 0.3353 | 0.6788 | 0.1971 | 0.0576 |
| Diseased vs Non-diseased | | 0.9834 | 0.1233 | 0.2362 | 0.1954 |
|  | Farm A - Diseased vs Non-diseased | 0.9768 | 0.1077 | 0.2874 | 0.1977 |
|  | Farm B - Diseased vs Non-diseased | 0.9040 | 0.6568 | 0.7161 | 0.6595 |
| Pre-SD and Post-SD Non-diseased pigs | | | | | |
| Farm A vs Farm B | | 0.1087 | 0.7919 | 0.2123 | 0.6062 |
| Sampling_1 vs Sampling_2 | | 0.2735 | 0.8655 | 0.7578 | 0.9999 |
| Sampling_1 vs Sampling_3 | | 0.4536 | 0.6619 | 0.2716 | 0.6678 |
| Sampling_1 vs Sampling_4 | | 0.4251 | 0.0023 ** | 0.5137 | 0.0025 ** |
| Sampling_2 vs Sampling_3 | | 0.9210 | 0.4196 | 0.4874 | 0.6281 |
| Sampling_2 vs Sampling_4 | | 0.0127 * | 0.0002 *** | 0.9247 | 0.0008 *** |
| Sampling_3 vs Sampling_4 | | 0.1239 | 0.6177 | 0.7075 | 0.6259 |
| Pre-SD vs Post-SD | | 0.4261 | 0.1527 | 0.5984 | 0.0874 |
|  | Farm A - Pre-SD vs Post-SD | 0.2774 | 0.0337 * | 0.5533 | 0.0226 * |
|  | Farm B - Pre-SD vs Post-SD | 0.4790 | 0.7819 | 0.9972 | 0.9442 |
| Pre-SD and Post-SD Diseased pigs | | | | | |
| Farm A vs Farm B | | 0.7700 | 0.4868 | 0.8600 | 0.6745 |
| Sampling_1 vs Sampling_2 | | 1.0000 | 0.9552 | 1.0000 | 0.8984 |
| Sampling_1 vs Sampling_3 | | 1.0000 | 0.9887 | 1.0000 | 0.9774 |
| Sampling_1 vs Sampling_4 | | 1.0000 | 0.9276 | 1.0000 | 0.8627 |
| Sampling_2 vs Sampling_3 | | 0.9200 | 0.9999 | 1.0000 | 0.9999 |
| Sampling_2 vs Sampling_4 | | 0.3000 | 0.9974 | 0.9900 | 0.9967 |
| Sampling_3 vs Sampling_4 | | 1.0000 | 1.0000 | 1.0000 | 1.0000 |
| Pre-SD vs Post-SD | | 0.0330 * | 0.2660 | 0.2200 | 0.2124 |
|  | Farm A - Pre-SD vs Post-SD | 0.0999 | 0.4627 | 0.4884 | 0.4428 |
|  | Farm B - Pre-SD vs Post-SD | 0.4000 | 0.4709 | 0.4000 | 0.4133 |

* *P* ≤ 0.05, ** *P* ≤ 0.01, *** *P* ≤ 0.001, **** *P* ≤ 0.0001
