# Supplementary material for: From predisposition to recovery: field evidence of interactions between the gut microbiota and Brachyspira hyodysenteriae infection
Source: Vet Res. 2026 Jan 30;57:25. doi: 10.1186/s13567-025-01646-1 (PMC12857038; doi:10.1186/s13567-025-01646-1)
Supplement: Supplementary file 6 — Additional file 6. Taxonomic beta diversity analyses. Influence of different factors on the ordination of samples and results of the multivariate permutational analysis of variance in the Bray-Curtis distances of the ordination. [file 13567_2025_1646_MOESM6_ESM.docx]

**Additional file 6** Taxonomic beta-diversity analyses. Influence of different factors on the ordination of samples and results of the multivariate permutational analysis of variance in the Bray-Curtis distances of the ordination.

| Factor | envfit | | PERMANOVA | |
| --- | --- | --- | --- | --- |
|  | R2 | *P*-value | R2 | *P*-value |
| Sampling 1 | | | | |
| Farm | 0.5026 | 0.001 *** | 0.2543 | 0.001 *** |
| Disease | 0.0086 | 0.831 | 0.0437 | 0.187 |
| Farm:Disease |  |  | 0.08325 | 0.011 * |
| Farm A | | | | |
| Disease | 0.0589 | 0.532 | 0.0986 | 0.225 |
| Farm B | | | | |
| Disease | 0.2195 | 0.211 | 0.2081 | 0.075 |
| Pre-SD sampling | | | | |
| Farm | 0.3486 | 0.001 *** | 0.1639 | 0.001 *** |
| Sampling | 0.0742 | 0.086 | 0.0418 | 0.032 * |
| Disease | 0.0303 | 0.347 | 0.0371 | 0.045 * |
| Farm:Sampling |  |  | 0.0535 | 0.009 ** |
| Farm:Disease |  |  | 0.0595 | 0.006 ** |
| Sampling:Disease |  |  | 0.0266 | 0.212 |
| Farm:Sampling:Disease |  |  | 0.0480 | 0.047 * |
| Farm A | | | | |
| Sampling | 0.1278 | 0.060 | 0.1204 | 0.006 ** |
| Disease | 0.0085 | 0.857 | 0.0647 | 0.136 |
| Sampling:Disease |  |  | 0.0352 | 0.596 |
| Farm B | | | | |
| Sampling | 0.2654 | 0.026 * | 0.1125 | 0.051 |
| Disease | 0.2278 | 0.049 * | 0.1509 | 0.017 * |
| Sampling:Disease |  |  | 0.1305 | 0.132 |
| Clinical SD sampling | | | | |
| Farm | 0.2763 | 0.001 *** | 0.1125 | 0.001 *** |
| Sampling | 0.4763 | 0.001 *** | 0.2194 | 0.001 *** |
| Disease | 0.0786 | 0.040 * | 0.0421 | 0.010 ** |
| Farm:Disease |  |  | 0.0268 | 0.152 |
| Sampling:Disease |  |  | 0.0775 | 0.004 ** |
| Farm A | | | | |
| Sampling | 0.3568 | 0.001 *** | 0.2488 | 0.001 *** |
| Disease | 0.0791 | 0.160 | 0.0470 | 0.179 |
| Sampling:Disease |  |  | 0.0501 | 0.142 |
| Farm B | | | | |
| Sampling | 0.3474 | 0.015 * | 0.2444 | 0.002 ** |
| Disease | 0.2414 | 0.043 * | 0.1370 | 0.003 ** |
| Sampling:Disease |  |  | 0.1594 | 0.004 ** |
| Post-SD sampling | | | | |
| Farm | 0.1827 | 0.001 *** | 0.1438 | 0.001 *** |
| Sampling | 0.1376 | 0.003 ** | 0.0795 | 0.001 *** |
| Disease | 0.0320 | 0.307 | 0.030 | 0.084 |
| Farm:Sampling |  |  | 0.0675 | 0.001 *** |
| Farm:Disease |  |  | 0.0272 | 0.119 |
| Sampling:Disease |  |  | 0.0332 | 0.068 |
| Farm:Sampling:Disease |  |  | 0.0289 | 0.105 |
| Farm A | | | | |
| Sampling | 0.2917 | 0.001 *** | 0.1354 | 0.001 *** |
| Disease | 0.0729 | 0.199 | 0.0534 | 0.119 |
| Sampling:Disease |  |  | 0.0821 | 0.008 ** |
| Farm B | | | | |
| Sampling | 0.4046 | 0.001 *** | 0.2480 | 0.001 *** |
| Disease | 0.1089 | 0.251 | 0.0779 | 0.218 |
| Sampling:Disease |  |  | 0.0561 | 0.523 |
| Sampling 4 | | | | |
| Farm | 0.3635 | 0.001 *** | 0.3405 | 0.001 *** |
| Disease | 0.0216 | 0.619 | 0.0242 | 0.518 |
| Farm:Disease |  |  | 0.0210 | 0.643 |
| Farm A |  |  |  |  |
| Disease | 0.137 | 0.122 | 0.0550 | 0.683 |
| Farm B |  |  |  |  |
| Disease | 0.043 | 0.799 | 0.1063 | 0.616 |
| Pre-SD and Post-SD Non-diseased pigs | | | | |
| Farm | 0.4547 | 0.001 *** | 0.1680 | 0.001 *** |
| Sampling | 0.1891 | 0.215 | 0.2171 | 0.001 *** |
| Dysentery | 0.0737 | 0.205 | 0.0248 | 0.679 |
| Farm:Sampling |  |  | 0.1355 | 0.008 ** |
| Farm:Dysentery |  |  | 0.034 | 0.394 |
| Farm A |  |  |  |  |
| Sampling | 0.5912 | 0.006 ** | 0.3698 | 0.001 *** |
| Dysentery | 0.3184 | 0.010 ** | 0.0388 | 0.740 |
| Farm B |  |  |  |  |
| Sampling | 0.8530 | 0.0167 * | 0.6101 | 0.0056 ** |
| Dysentery | 0.4624 | 0.1000 | 0.1613 | 0.2000 |
| Pre-SD and Post-SD Diseased pigs | | | | |
| Farm | 0.2466 | 0.004 ** | 0.1407 | 0.001 *** |
| Sampling | 0.1676 | 0.211 | 0.1686 | 0.091 |
| Dysentery | 0.0791 | 0.168 | 0.0318 | 0.493 |
| Farm:Sampling |  |  | 0.1764 | 0.020 * |
| Farm:Dysentery |  |  | 0.0482 | 0.144 |
| Farm A |  |  |  |  |
| Sampling | 0.2168 | 0.409 | 0.2689 | 0.024 * |
| Dysentery | 0.1838 | 0.050 | 0.1070 | 0.028 * |
| Farm B |  |  |  |  |
| Sampling | 0.1614 | 0.800 | 0.5420 | 0.278 |
| Dysentery | 0.0807 | 0.900 | 0.0795 | 0.856 |

* *P* ≤ 0.05, ** *P* ≤ 0.01, *** *P* ≤ 0.001, **** *P* ≤ 0.0001
