# Supplementary material for: From predisposition to recovery: field evidence of interactions between the gut microbiota and Brachyspira hyodysenteriae infection
Source: Vet Res. 2026 Jan 30;57:25. doi: 10.1186/s13567-025-01646-1 (PMC12857038; doi:10.1186/s13567-025-01646-1)
Supplement: Supplementary file 7 — Additional file 7. Functional beta diversity analyses. Influence of different factors on the ordination of samples and results of the multivariate permutational analysis of variance in the Bray-Curtis distances of the ordination. [file 13567_2025_1646_MOESM7_ESM.docx]

**Additional file 7** Functional beta-diversity analyses. Influence of different factors on the ordination of samples and results of the multivariate permutational analysis of variance in the Bray-Curtis distances of the ordination.

| Factor | envfit | | PERMANOVA | |
| --- | --- | --- | --- | --- |
|  | R2 | *P*-value | R2 | *P*-value |
| Sampling 1 | | | | |
| Farm | 0.2122 | 0.004 ** | 0.2019 | 0.001 *** |
| Disease | 0.0082 | 0.879 | 0.0398 | 0.306 |
| Farm:Disease |  |  | 0.0799 | 0.049 * |
| Farm A |  |  |  |  |
| Disease | 0.0578 | 0.526 | 0.1089 | 0.146 |
| Farm B |  |  |  |  |
| Disease | 0.1145 | 0.479 | 0.1637 | 0.240 |
| Pre-SD sampling | | | | |
| Farm | 0.2016 | 0.001 *** | 0.1708 | 0.001 *** |
| Sampling | 0.0260 | 0.419 | 0.0222 | 0.421 |
| Disease | 0.0140 | 0.664 | 0.0346 | 0.131 |
| Farm:Sampling |  |  | 0.0263 | 0.314 |
| Farm:Disease |  |  | 0.0508 | 0.060 |
| Sampling:Disease |  |  | 0.0158 | 0.612 |
| Farm:Sampling:Disease |  |  | 0.0342 | 0.178 |
| Farm A |  |  |  |  |
| Sampling | 0.0100 | 0.852 | 0.0518 | 0.358 |
| Disease | 0.0652 | 0.231 | 0.0516 | 0.342 |
| Sampling:Disease |  |  | 0.0503 | 0.346 |
| Farm B |  |  |  |  |
| Sampling | 0.0429 | 0.625 | 0.0642 | 0.470 |
| Disease | 0.1186 | 0.234 | 0.1252 | 0.181 |
| Sampling:Disease |  |  | 0.0651 | 0.391 |
| Clinical SD sampling | | | | |
| Farm | 0.1089 | 0.032 * | 0.0853 | 0.002 ** |
| Sampling | 0.2745 | 0.001 *** | 0.1735 | 0.001 *** |
| Disease | 0.0457 | 0.194 | 0.0594 | 0.013 * |
| Farm:Disease |  |  | 0.0359 | 0.122 |
| Sampling:Disease |  |  | 0.0451 | 0.363 |
| Farm A |  |  |  |  |
| Sampling | 0.2285 | 0.006 ** | 0.1744 | 0.001 *** |
| Disease | 0.0187 | 0.651 | 0.0454 | 0.258 |
| Sampling:Disease |  |  | 0.0358 | 0.396 |
| Farm B |  |  |  |  |
| Sampling | 0.2723 | 0.035 * | 0.2161 | 0.009 ** |
| Disease | 0.2952 | 0.043 * | 0.2060 | 0.016 * |
| Sampling:Disease |  |  | 0.0729 | 0.317 |
| Post-SD sampling | | | | |
| Farm | 0.1936 | 0.001 *** | 0.1170 | 0.001 *** |
| Sampling | 0.0878 | 0.037 * | 0.0791 | 0.001 *** |
| Disease | 0.0346 | 0.290 | 0.0194 | 0.366 |
| Farm:Sampling |  |  | 0.0711 | 0.002 ** |
| Farm:Disease |  |  | 0.0230 | 0.277 |
| Sampling:Disease |  |  | 0.3260 | 0.116 |
| Farm:Sampling:Disease |  |  | 0.0760 | 0.003 ** |
| Farm A |  |  |  |  |
| Sampling | 0.0364 | 0.428 | 0.0713 | 0.082 |
| Disease | 0.0318 | 0.473 | 0.0266 | 0.696 |
| Sampling:Disease |  |  | 0.1303 | 0.003 ** |
| Farm B |  |  |  |  |
| Sampling | 0.3669 | 0.009 ** | 0.3023 | 0.001 *** |
| Disease | 0.0912 | 0.301 | 0.0670 | 0.224 |
| Sampling:Disease |  |  | 0.1139 | 0.099 |
| Sampling 4 | | | | |
| Farm | 0.4687 | 0.001 *** | 0.3316 | 0.001 *** |
| Disease | 0.0146 | 0.711 | 0.0154 | 0.789 |
| Farm:Disease |  |  | 0.022 | 0.580 |
| Farm A |  |  |  |  |
| Disease | 0.0407 | 0.574 | 0.0471 | 0.692 |
| Farm B |  |  |  |  |
| Disease | 0.0432 | 0.714 | 0.0797 | 0.636 |
| Pre-SD and Post-SD Non-diseased pigs | | | | |
| Farm | 0.2482 | 0.006 ** | 0.1601 | 0.002 ** |
| Sampling | 0.3251 | 0.038 * | 0.2581 | 0.003 ** |
| Dysentery | 0.1124 | 0.073 | 0.0264 | 0.558 |
| Farm:Sampling |  |  | 0.1291 | 0.052 |
| Farm:Dysentery |  |  | 0.0156 | 0.834 |
| Farm A |  |  |  |  |
| Sampling | 0.4628 | 0.045 * | 0.3448 | 0.031 * |
| Dysentery | 0.1527 | 0.092 | 0.0300 | 0.838 |
| Farm B |  |  |  |  |
| Sampling | 0.8478 | 0.017 * | 0.7556 | 0.011 * |
| Dysentery | 0.2244 | 0.400 | 0.0696 | 0.639 |
| Pre-SD and Post-SD Diseased pigs | | | | |
| Farm | 0.1645 | 0.016 * | 0.1305 | 0.002 ** |
| Sampling | 0.1055 | 0.563 | 0.1346 | 0.139 |
| Dysentery | 0.0636 | 0.253 | 0.0333 | 0.415 |
| Farm:Sampling |  |  | 0.2052 | 0.014 * |
| Farm:Dysentery |  |  | 0.0697 | 0.080 |
| Farm A |  |  |  |  |
| Sampling | 0.1800 | 0.526 | 0.2243 | 0.237 |
| Dysentery | 0.0317 | 0.639 | 0.1450 | 0.031 * |
| Farm B |  |  |  |  |
| Sampling | 0.5630 | 0.167 | 0.5326 | 0.217 |
| Dysentery | 0.1565 | 0.700 | 0.098 | 0.733 |

* *P* ≤ 0.05, ** *P* ≤ 0.01, *** *P* ≤ 0.001, **** *P* ≤ 0.0001
