# Supplementary material for: From predisposition to recovery: field evidence of interactions between the gut microbiota and Brachyspira hyodysenteriae infection
Source: Vet Res. 2026 Jan 30;57:25. doi: 10.1186/s13567-025-01646-1 (PMC12857038; doi:10.1186/s13567-025-01646-1)
Supplement: Supplementary file 8 — Additional file 8. Results of microbiota linear models for differential abundance species and functions. [file 13567_2025_1646_MOESM8_ESM.docx]

**Additional file 8** Results of microbiota linear models for differential abundance species and functions.

| Specie/Function | baseMean | log2FoldChange | lfcSE | stat | *P*-value | *P* adj | df |
| --- | --- | --- | --- | --- | --- | --- | --- |
| Species sampling 1 | | | | | | | |
| Farm B | | | | | | | |
| Roseburia_sp_499 | 202.2174 | -6.0068 | 0.5241 | -11.4623 | 8.6438e-06 | 0.0021 | 7 |
| Species Pre-SD sampling | | | | | | | |
| UBA4363.sp017937025 | 12478.2224 | -1.0248 | 0.2553 | -4.0137 | 3.3034e-04 | 0.0357 | 32 |
| UBA2868.sp004552595 | 8492.1870 | -1.8253 | 0.4655 | -3.9210 | 4.2082e-04 | 0.0357 | 33 |
| Ruminiclostridium_E;s | 5055.0776 | -1.3596 | 0.3563 | -3.8160 | 5.8263e-04 | 0.0395 | 32 |
| Prevotella_sp_P3_92 | 30852.5248 | -1.7955 | 0.4823 | -3.7229 | 7.3341e-04 | 0.0414 | 33 |
| Prevotella_sp_P5_92 | 15207.4428 | -0.7743 | 0.1888 | -4.1011 | 2.6177e-04 | 0.0357 | 32 |
| Treponema_rectale | 870.6731 | -1.6751 | 0.3549 | -4.7206 | 4.1917e-05 | 0.0142 | 33 |
| Farm B |  |  |  |  |  |  |  |
| Eubacterium_coprostanoligenes | 822.901 | 2.0746 | 0.3853 | 5.3839 | 1.6431e-04 | 0.0236 | 12 |
| Treponema_rectale | 1278.799 | -3.5196 | 0.4146 | -8.4882 | 2.0398e-06 | 0.0006 | 12 |
| Species Clinical SD sampling | | | | | | | |
| Egerieousia.sp004561775 | 9356.0156 | 1.3455 | 0.3742 | 3.5958 | 1.0425e-03 | 0.0351 | 33 |
| UBA3839.sp900314125 | 36.4359 | 3.5106 | 1.0366 | 3.3868 | 1.8424e-03 | 0.0442 | 33 |
| Limimorpha.sp900769945 | 35482.2860 | 1.4358 | 0.4044 | 3.5503 | 1.2630e-03 | 0.0351 | 31 |
| Mucispirillum.sp910586745 | 75.9402 | 2.0435 | 0.4735 | 4.3155 | 1.3630e-04 | 0.0079 | 33 |
| UBA2658.sp018385135 | 59.4861 | 3.5515 | 1.0075 | 3.5252 | 1.3406e-03 | 0.0351 | 31 |
| ER4.sp900317525 | 10.7554 | 3.3218 | 0.7978 | 4.1635 | 2.2620e-04 | 0.0109 | 31 |
| RUG369;s | 90.6259 | 1.8599 | 0.4788 | 3.8843 | 4.7465e-04 | 0.0195 | 33 |
| Prevotella_sp_P3_92 | 7996.5911 | -2.3167 | 0.6535 | -3.5452 | 1.1977e-03 | 0.0351 | 33 |
| Inconstantimicrobium_porci | 2606.5902 | -5.2879 | 0.9809 | -5.3908 | 6.3230e-06 | 0.0018 | 32 |
| Acetivibrio_ethanolgignens | 190.6690 | 2.6620 | 0.5852 | 4.5490 | 6.9211e-05 | 0.0058 | 33 |
| Dysosmobacter_sp_BX15 | 8.0394 | 1.9522 | 0.4335 | 4.5038 | 8.0184e-05 | 0.0058 | 33 |
| Brachyspira_hyodysenteriae | 231.0766 | 2.7255 | 0.5545 | 4.9155 | 2.6945e-05 | 0.0039 | 31 |
| Functions Clinical SD sampling | | | | | | | |
| Urea decomposition | 433.1411 | -0.4814 | 0.1096 | -4.3913 | 1.2184e-04 | 0.0213 | 31 |
| Bacteroides capsular polysaccharide transcription antitermination proteins | 60.9108 | 0.3997 | 0.0904 | 4.4207 | 1.0052e-04 | 0.0213 | 33 |
| Experimental ThMP RZ | 255.4701 | -0.2147 | 0.0469 | -4.5768 | 7.3049e-05 | 0.0213 | 31 |
| Pterin carbinolamine dehydratase | 3.3804 | 0.5816 | 0.1228 | 4.7382 | 3.9816e-05 | 0.0162 | 33 |
| Coenzyme PQQ synthesis | 4.3709 | 0.6651 | 0.1545 | 4.3056 | 1.5175e-04 | 0.0231 | 31 |
| Th552 | 48.5510 | -0.3489 | 0.0798 | -4.3709 | 1.2233e-04 | 0.0213 | 32 |
| Synthesiis of osmoregulatedperiplasmic glucans | 583.7464 | -0.2696 | 0.0513 | -5.2558 | 8.6739e-06 | 0.0106 | 33 |
| Taurine Utilization | 24.8057 | -0.4221 | 0.0877 | -4.8107 | 3.2190e-05 | 0.0162 | 33 |
| Species Sampling 4 | | | | | | | |
| Farm B |  |  |  |  |  |  |  |
| CAG-533.sp000434495 | 792.706 | 1.7909 | 0.2253 | 7.9477 | 9.5046e-05 | 0.0179 | 7 |
| Functions Sampling 4 | | | | | | | |
| Farm B |  |  |  |  |  |  |  |
| Denitrification | 103.3648 | 0.2163 | 0.0215 | 10.0793 | 2.0309e-05 | 0.0243 | 7 |
| Species Pre-SD and Post-SD Non-diseased pigs | | | | | | | |
| Farm B |  |  |  |  |  |  |  |
| UBA3663.sp016293065 | 12.9452 | 9.5774 | 0.7425 | 12.8983 | 0.0002 | 0.0204 | 4 |
| Anaerovibrio_slackiae | 23786.6007 | -1.6120 | 0.1164 | -13.8490 | 0.0002 | 0.0204 | 4 |
| Species Pre-SD and Post-SD Diseased pigs | | | | | | | |
| Treponema_D.sp018385315 | 12009 | 1.3332 | 0.2312 | 5.7665 | 1.4791e-05 | 0.0035 | 19 |
| Farm A |  |  |  |  |  |  |  |
| Alloprevotella.sp004552865 | 3221.4254 | -2.5893 | 0.5435 | -4.7645 | 0.0004 | 0.0243 | 13 |
| UMGS1470.sp900552105 | 18.4898 | 3.9752 | 0.7052 | 5.6372 | 0.0001 | 0.0198 | 12 |
| Treponema_D.sp018385315 | 7924.5863 | 1.2111 | 0.2286 | 5.2986 | 0.0002 | 0.0235 | 11 |
